# Pulmonary Toxicity, Distribution, and Clearance of Intratracheally Instilled Silicon Nanowires in Rats

**DOI:** 10.1155/2012/398302

**Published:** 2012

**Authors:** Jenny R. Roberts, Robert R. Mercer, Rebecca S. Chapman, Guy M. Cohen, Sarunya Bangsaruntip, Diane Schwegler-Berry, James F. Scabilloni, Vincent Castranova, James M. Antonini, Stephen S. Leonard

**Affiliations:** 1 Health Effects Laboratory Division, National Institute for Occupational Safety and Health, 1095 Willowdale Road, Morgantown, WV 26505, USA; 2 T.J. Watson Research Center, IBM, Route 134, P.O. Box 218, Yorktown Heights, NY 10598, USA

## Abstract

Silicon nanowires (Si NWs) are being manufactured for use as sensors and transistors for circuit applications. The goal was to assess pulmonary toxicity and fate of Si NW using an *in vivo* experimental model. Male Sprague-Dawley rats were intratracheally instilled with 10, 25, 50, 100, or 250 *μ*g of Si NW (~20–30 nm diameter; ~2–15 *μ*m length). Lung damage and the pulmonary distribution and clearance of Si NW were assessed at 1, 3, 7, 28, and 91 days after-treatment. Si NW treatment resulted in dose-dependent increases in lung injury and inflammation that resolved over time. At day 91 after treatment with the highest doses, lung collagen was increased. Approximately 70% of deposited Si NW was cleared by 28 days with most of the Si NW localized exclusively in macrophages. In conclusion, Si NW induced transient lung toxicity which may be associated with an early rapid particle clearance; however, persistence of Si NW over time related to dose or wire length may lead to increased collagen deposition in the lung.

## 1. Introduction

It has been predicted that nanotechnology could have an impact as large as $1 trillion on the global economy over the next decade, and that as many as two million workers could be employed in this field [1]. The aerodynamic parameters and properties of nanomaterials in the form of dusts or aerosols mean that respiratory exposure in the workplace is a great concern. Engineered nanomaterials are defined as materials intentionally produced to have at least one dimension that is less than 100 nm. Toxic effects attributed to engineered nanomaterial vary depending on the physicochemical properties of the particular material, including size, shape, surface area, composition, and reactivity.

Fiber- or rod-shaped nanomaterials with high aspect ratio, the ratio of length to diameter, are of particular concern in respiratory toxicology due to their resemblance in shape to toxic fibers, such as asbestos [2]. Even materials considered to be relatively low in pulmonary toxicity in relationship to composition, such as titanium or carbon, can pose a greater risk of pulmonary toxicity as a high aspect ratio nanomaterial. Pulmonary toxicity associated with TiO_2_ nanobelts has been shown to be size and shape dependent. For example, pulmonary inflammation and lung injury in rats associated with short anatase titanium rods (200 nm ***×*** 35 nm) [3] or short rutile TiO_2_ nanorods (20 nm ***×*** 40 nm) were shown to be very transient [4]; however, TiO_2_ nanobelts greater than 15 *μ*m were shown to cause greater toxicity *in vivo* and *in vitro* when compared to wires less than 5 *μ*m in length [5]. Perhaps the best and most studied examples of nanomaterials with high aspect ratio are single-walled (SW) and multiwalled (MW) carbon nanotubes (CNT), for which respiratory exposure in animal models can result in pleural penetration and fibrosis [6–11].

As the field of nanotechnology develops, the manufacture of high aspect ratio silicon (Si) nanowires (NWs) is increasing with the ability to utilize their semiconductive, thermal, optical, mechanical, and chemical properties in a multitude of devices and applications [12]. Si NWs are anisotropic filamentary crystals of silicon with high aspect ratio, typically synthesized by a chemical vapor deposition (CVD) method and assisted by a metallic catalyst (typically gold nanoparticles) where at a given temperature time controls the length of the crystal rod and the size of the catalyst controls the diameter. Applications for which Si NW are being employed include, but are not limited to, batteries and energy storage, solar cells, catalysts, gas sensors, and biological applications such as drug delivery systems, gene delivery systems, *in vivo* imaging agents, and biosensors [12, 13].

Si, a relatively nontoxic crustal element as a semiconductor, is one of the most abundant elements in nature. Although pure Si is uncommon and usually exists as a form of silicon dioxide or as a mineral composite silicate, free Si is commonly used in a number of different manufacturing industries. Currently, Si as dust is considered to be a relatively inert nuisance dust, with the National Institute for Occupational Safety and Health (NIOSH) recommended exposure limit (REL) time weighted average (TWA) set at 5 mg/m^3^ for respirable dust (particles <10 *μ*m in diameter) and 10 mg/m^3^ for total dust [14]. Although numerous studies have emerged that investigated toxicity of crystalline and amorphous nanosized silicon dioxide [15], studies on toxicity of nanosized silicon of any shape are few. There are only a small number of studies that have examined the potential toxicity of Si NW *in vitro*. These studies were primarily related to applications of vertically oriented Si NW arrays on Si substrates where osteoblasts [16], embryonic kidney cells [17, 18], or hepatocyte cell lines [19] were added to the arrays to monitor cytotoxicity, adhesion, proliferation, and spreading for potential use in tissue engineering and drug/gene delivery applications. There are no studies that address toxicity of Si NW *in vivo* and there are currently no data available on toxicity that associated pulmonary exposure with Si NW.

The goal of the current study was to characterize potential toxicity following pulmonary exposure to Si NW *in vivo* using an animal model. Single-crystal silicon NWs were synthesized by IBM T.J. Watson Research Center using the vapor-liquid-solid method in an ultrahigh vacuum-CVD chamber with silane as the silicon precursor and gold as the catalyst. The wires had a diameter in the range of ~20–30 nm diameter and length was ~15 *μ*m. Each wire had a very uniform diameter, with less than 1 nm change along the length of a wire. A 25 nm gold nanoparticle was present. This particle catalyzed the growth of the wire in the form of a Au-Si eutectic particle. NWs were isolated from the wafer upon which they grew, suspended in a physiologic dispersion medium (DM) [20], sonicated, and characterized for length and reactivity. A dose-response time course study was conducted using Sprague-Dawley rats that were intratracheally instilled with doses of Si NW in DM ranging from 10 to 250 *μ*g per rat. Parameters of lung toxicity and disease, including lung injury, inflammation, and fibrotic responses, were evaluated at time points ranging from 1 to 91 days after-exposure.

## 2. Methods

### 2.1. Silicon (Si) Nanowire (NW) Synthesis, Characterization, and Preparation for In Vitro and In Vivo Studies

#### 2.1.1. Nanowire Synthesis

Si NWs were synthesized at IBM T.J. Watson Research Center in an ultrahigh vacuum chemical vapor deposition (CVD) chamber using a vapor-liquid-solid (VLS) method with silane as the silicon precursor and 25 nm gold nanoparticles as the catalyst. Briefly, a 2 nm thin gold film is deposited on a clean silicon (111) surface and annealed at 450°C so that the gold film agglomerates into nanoparticles with an average diameter of ~25 nm. When the growth is initiated, a metallic-silicon liquid alloy is formed (e.g., Au-Si eutectic). With additional supply of Si from the gas phase (e.g., SiH_4_), the metallic-silicon droplet becomes supersaturated with Si, and the excess silicon is deposited at the solid-liquid interface. As a result, the liquid droplet rises from the original substrate surface to the tip of a growing silicon nanowire crystal. Growth temperature is kept below the decomposition temperature of the Si precursor (about 500°C when SiH_4_ is used) so that no deposition of silicon take places on the nanowire sidewalls (i.e., no radial growth). As a result the only growth taking place is that enabled by the metallic catalyst which leads to anisotropic growth. Growth was targeted to produce Si NW ~15 *μ*m in length and 25 nm in diameter with Au at one end [21, 22].

#### 2.1.2. Nanowire Isolation and Dispersion

Wafers with grown Si NWs were handled in a hood during the isolation and dispersion process to ensure sterility. The wafers were cleaved and sonicated in ethanol. The wafer was removed, an aliquot of the ethanol containing the Si NW was transferred to a filter, the ethanol was evaporated, and a weight/volume measurement was made. The ethanol was evaporated from the sample at 60°C, and the Si NWs were resuspended in sterile phosphate-buffered saline (PBS). Si NWs were imaged on a field emission scanning electron microscope (FESEM, Hitachi Model S-4800) and were found to aggregate and form bundles in PBS. Therefore, prior to characterization and *in vivo* studies, Si NWs were suspended in a sterile PBS-based dispersion medium (DM) consisting of 0.6 mg/mL rat serum albumin +0.01 mg/mL dipalmitoyl phosphatidylcholine (DPPC) and sonicated at 10 watts for 5 minutes. This DM has been shown to be nontoxic *in vivo* and does not mask inherent particle toxicity [20].

#### 2.1.3. Elemental Analysis for Au

Isolated dispersed Si NWs were analyzed for Au content by North Carolina State University Nuclear Reactor Program, Department of Nuclear Engineering (Raleigh, NC) using neutron activation. Briefly, samples and standards were irradiated in a PULSTAR reactor rotating exposure port for 12 minutes. Samples decayed for approximately one week and were counted for 1 hour each on a gamma spectroscopy system analyzing for Au.

#### 2.1.4. Electron Spin Resonance (ESR)

The generation of the hydroxyl radicals on the particle surface as an indicator of surface reactivity was evaluated by ESR using an EMX spectrometer (Bruker Instruments Inc., Billerica, MA) and a flat cell assembly. This technique involved the addition-type reaction of a short-lived radical with a diamagnetic compound (spin trap) to form a relatively long-lived free radical product (the spin adduct) which can be observed by conventional ESR [23]. For this study, hydroxyl radical was generated from a Fenton-like reaction system after the Si NW sample suspended in dispersion media (100 mg) or silica (Min-U-Sil a-quartz, US Silica Co., Berkeley Springs, WV; positive control; 100 mg) was combined with H_2_O_2_ [1 mM] in the presence of 100 mM of the spin trap 5,5-dimethyl-1-pyrroline N-oxide (DMPO) and PBS to a final volume of 1 mL. An additional sample of Si NW (100 mg) was also prepared and examined after etching processes which removes the gold catalyst to assess whether gold played a role in surface reactivity. Reactions were allowed to incubate 3 min at room temperature before measurement and then transferred to a flat cell for ESR measurement. Signal intensity of the spin adduct, which corresponds to the amount of a given radical species, was determined by integration of the characteristic wave form for that radical. The wave form was then measured and quantified.

#### 2.1.5. NW Length Distribution

Twenty micrographs of Si NW suspended and sonicated in DM were imaged using FESEM at 5 to 20 kV. A total of 730 nanowires were counted, and the length of each was measured using Gundersen’s unbiased counting rules [24] to obtain the length frequency and distribution. In addition, micrographs of Si NW before and after sonication in DM were compared to assess potential wire breakage due to preparation of the NW suspension.

### 2.2. Animals

Male Sprague-Dawley [Hla: (SD) CVF] (SD) rats from Hilltop Lab Animals (Scottdale, PA), weighing 250–300 g and free of viral pathogens, parasites, mycoplasmas, *Helicobacter*, and CAR *Bacillus*, were used for all exposures. The rats were acclimated for at least 6 days after arrival and were housed in ventilated polycarbonate cages on Alpha-Dri cellulose chips and hardwood Beta chips as bedding, and provided HEPA-filtered air, irradiated Teklad 2918 diet, and tap water *ad libitum*. The animal facilities are specific pathogen free, environmentally controlled, and accredited by the Association for Assessment and Accreditation of Laboratory Animal Care International (AAALAC). All animal procedures used during the study were reviewed and approved by the National Institute for Occupational Safety and Health Animal Care and Use Committee.

### 2.3. In Vitro Uptake by Primary Alveolar Macrophages (AMs)

Bronchoalveolar lavage (BAL) was performed by washing the lungs of the naïve rats with aliquots of PBS in order to obtain primary AMs. Briefly, rats were euthanized with an overdose of sodium pentobarbital (*>*100 mg/kg body weight; Sleepaway, Fort Dodge Animal Health, Wyeth, Madison, NJ), the trachea was cannulated, the chest cavity was opened, and BAL was performed on the lungs via the tracheal cannula. Five washes of 6 mL each of PBS were performed while massaging the chest cavity. The collected fluid was then centrifuged and the cell pellet was resuspended in 1 mL PBS. Cells were counted using a Coulter Multisizer II (Coulter Electronics, Hialeah, FL) to determine the total number of AMs recovered from the lavage. Based on BAL cell counts, 2.5 ***×*** 10^5^ AMs from each rat were placed in RPMI 1640 culture media without serum (Sigma-Aldrich Co., St. Louis, MO) for 1.5 hr before treatment with 25 *μ*g of Si NW. After incubation for 4 hours, the cells were washed with PBS, fixed in 10% neutral buffered formalin and prepared for FESEM to demonstrate AM uptake of Si NW.

### 2.4. In Vivo Exposure and Study Design

On day 0, male Sprague-Dawley rats were lightly anesthetized by an intra-peritoneal injection of 0.6 mL of a 1% solution of sodium methohexital (Brevital, Eli Lilly, Indianapolis, IN) and intratracheally instilled with 10, 25, 50, 100, or 250 *μ*g of Si NW in DM. Vehicle control rats received an equivalent volume (300 *μ*L) of sterile DM by intratracheal instillation. Rats were humanely euthanized 1, 3, 7, 28, and 91 days after-exposure (*n* ***=*** 4/dose/time point). Bronchoalveolar lavage (BAL) was performed on the right lungs and BAL cells (BALCs) and fluid (BALF) were retained for analysis. Lung injury and inflammation were evaluated as the presence of lactate dehydrogenase (LDH) activity, albumin, cytokines, and chemokines in BALF. BALCs were centrifuged onto slides, stained, and differentials were determined. Oxidant/free radical production by BALC was measured by chemiluminescence to further evaluate the inflammatory response. After lavage, the right lung lobes (apical, cardiac, and azygous) were weighed and preserved for total collagen content assays to estimate fibrotic changes in the lung. The left lung was preserved for histopathological analysis of injury, inflammation and disease, and morphometric analysis of Si NW clearance and tissue distribution (airways versus alveolar region and within the alveolar region in macrophages, tissue, or airspace). As an indicator of fibrotic disease, morphometric analysis of fibrillar collagen content was also performed.

### 2.5. BAL Cell and Fluid Collection

BAL was performed at each time point after exposure by washing the lungs of treated rats with aliquots of PBS in order to obtain pulmonary cells for morphological and functional analysis, and the acellular BALF was retained for analysis of indicators of tissue damage and cellular activity. Rats were euthanized with an overdose of sodium pentobarbital (*>*100 mg/kg body weight; Sleepaway, Fort Dodge Animal Health, Wyeth, Madison, NJ, USA), the trachea was cannulated, the chest cavity was opened, the left lung was clamped off, and BAL was performed on the right lung via the tracheal cannula at different time points after I.T. The acellular fraction of the first BAL was obtained by filling the right lung with 1 mL/100 g body weight of PBS, massaging for 30 seconds, withdrawing, and repeating the process one more time. This concentrated aliquot was withdrawn, retained, kept separately, and was designated as the first fraction of BALF. The following aliquots were 6 mL in volume, instilled once with light massaging, withdrawn, and combined until a 30 mL volume was obtained. For each animal, both fractions of BAL were centrifuged, the cell pellets were combined and resuspended in 1 mL of PBS, and the acellular fluid from the first fraction was retained for further analysis. After lavage, the right lobes were weighed and snap frozen for later analysis of collagen content.

### 2.6. Evaluation of Lung Injury, Inflammation, and Disease

#### 2.6.1. Analysis of Albumin and Lactate Dehydrogenase (LDH) Activity

The presence of LDH activity and albumin in the BALF of all treatment groups was measured at each time point after exposure to evaluate cytotoxicity and the loss of integrity of the alveolar-capillary barrier, respectively. Measurements of both albumin and LDH activity in the acellular fluid were obtained using a Cobas Mira analyzer (Roche Diagnostic Systems, Montclair, IN). Albumin was determined by spectrophotometric measurement of the reaction product of albumin with bromocresol green (628 nm) according to a method by Sigma Diagnostics (St. Louis, MO, USA). LDH activity was quantified by detection of the oxidation of lactate coupled to the reduction of NAD+ at a spectrophotometric setting of 340 nm.

#### 2.6.2. BALF Chemokine and Inflammatory and Immune Cytokine Analysis

Cytokines and chemokines involved in inflammatory and immune responses were measured at each time point after exposure in the BALF of rats treated with Si NW or DM using commercially available enzyme-linked immunosorbent assay (ELISA) kits (BioSource International Inc., Camarillo, CA, USA). The following cytokines and chemokines were quantified: tumor necrosis factor-*α* (TNF-*α*), transforming growth factor-*β* (TGF-*β*), interleukin (IL)-2, IL-4, IL-6, IL-10, IL-12p70, interferon-*γ* (IFN-*γ*), monocyte chemotactic protein (MCP)-1, and macrophage inflammatory protein (MIP)-2.

#### 2.6.3. BAL Cell Differentials and Particle Uptake by AMs

Total BAL cells collected from rats treated with Si NW or DM were counted using a Coulter Multisizer II (Coulter Electronics, Hialeah, FL). Cell differentials were performed to determine the total number of AMs, neutrophils, lymphocytes, and eosinophils. Briefly, 1 ***×*** 10^5^ cells from each rat were spun down onto slides with a Cytospin 3 centrifuge (Shandon Life Sciences International, Cheshire, England) and labeled with Leukostat stain (Fisher Scientific, Pittsburgh, PA, USA) to differentiate cell types. Two hundred cells per slide were counted, and the percentage of AMs, poly-morphonuclear cells (PMNs, neutrophils), lymphocytes, and eosinophils was multiplied by the total number of cells to calculate the total number of each cell type.

#### 2.6.4. Chemiluminescence (CL)

To measure the production of reactive oxidant species, CL was measured according to the method of Antonini et al. [25]. Luminol was used as an amplifier to enhance detection of the light, and 2 mg/mL of unopsonized zymosan (Sigma Chemical Company, St. Louis, MO, USA) or 3 *μ*M phorbol myristate acetate (PMA; Sigma Chemical Company, St. Louis, MO) was added to the assay immediately prior to the measurement of CL to activate the cells. Because rat PMNs do not respond to unopsonized zymosan, the zymosan-stimulated CL produced is from AMs, whereas the soluble stimulant, PMA, activates both PMNs and AMs to generate reactive oxidant species. Measurement of CL was done using an automated Berthold Auto-lumat LB 953 luminometer (Wallace, Inc., Gaithersburg, MD, USA) for 15 min, and the integral of counts per minute (cpm) versus time was calculated. The production of CL was calculated as the cpm of stimulated cells minus the cpm of the corresponding resting cells, then normalized to the total number of BAL AMs for zymosan-stimulated CL and total BAL cells for PMA-stimulated CL.

#### 2.6.5. Histopathology

The left lungs of Si NW-treated and control rats were fixed with 10% neutral buffered formalin by airway fixation at total lung capacity. The left lungs were embedded in paraffin, sectioned onto slides, and stained with hematoxylin and eosin (H&E). H&E-stained slides were quantitatively analyzed for indications of inflammation, injury, and fibrosis by a certified veterinary pathologist at Charles River Laboratories (Wilmington, MA, USA) who was blinded to the treatment groups. Indices of inflammation and injury were scored on scale of 0–5, where 0 = no observed effect, 1 = minimal response, 2 = mild response, 4 = moderate response, and 5 = severe response.

#### 2.6.6. Sircol Assay for Lung Collagen Content

Lung collagen content was determined by quantifying total soluble collagen using the sircol collagen assay kit (Accurate Chemical and Scientific, Westbury, NY, USA). The apical lobe was thawed then homogenized in 0.7 mL of 0.5 M acetic acid containing pepsin with a 1 : 10 ratio of pepsin to tissue wet weight. Each sample was stirred vigorously for 24 h at 4°C and centrifuged, and 200 *μ*L of supernatant was assayed according to the manufacturer’s instructions. Briefly, standards and lung samples were mixed with the dye reagent. The collagen-dye complex was centrifuged into a pellet, and the supernatant was discarded. The pellet was washed to remove unbound dye, centrifuged again, and supernatant discarded. The bound dye was released and dissolved via vortexing in the presence of the alkali reagent provided. The released dye was measured spectrophotometrically at 555 nm. The final concentration was normalized to the weight of the lung tissue.

### 2.7. Morphometric Lung Tissue Analysis

#### 2.7.1. Connective Tissue Thickness

Tissue sections from left lung of control rats and rats treated with 100 *μ*g Si NW were deparaffinized and stained with Sirius Red for detection of connective tissue, particularly fibrillar collagen and to enhance contrast between tissue and Si NW. Slides were immersed in 0.1% Picrosirius solution (100 mg of Sirius Red F3BA in 100 mL of saturated aqueous picric acid) for 2 hours followed by washing for 1 minute in 0.01 N HCl. They were then counterstained with hematoxylin for 2 minutes, dehydrated, and mounted with a coverslip for imaging. Tissue sections from left lung were deparaffinized, rehydrated, and stained with Sirius Red for detection of connective tissue and to enhance visualization of Si NW. Quantitative morphometric methods [6, 26] were used to measure the relative distribution of nanoparticles in airways, alveolar airspace, alveolar tissue, and alveolar macrophages. Quantitative morphometric methods were used to measure the average thickness of Sirius Red-positive connective tissue in the alveolar regions. Volume (% of the alveolar wall) and thickness were measured by standard morphometric analyses [27, 28]. This consisted of basic point and intercept counting. An eyepiece counting overlay consisting of 11 by 11 lines (121 total points for each throw of the overlay) was used with a 100X oil immersion objective. A grid pattern for throws of the counting overlay was used in order to insure a uniform sampling of the section which did not overweight interior points. The counting overlay throws of the eyepiece were positioned over the section at 12 uniformly spaced grid points in both X and Y coordinates. These 12 grid points were determined using the stage micrometer scale to measure the X and Y bounds of the section. Using the bounding rectangle of these coordinates a 3 by 4 grid was selected and the 12 intersections were used as the center point for each of the eyepiece counting overlay throws. Volume was determined by counting the number of points over the Sirius Red-positive connective tissues in the alveolar regions. Surface density of the alveolar wall was determined from intercepts between a line overlay and the alveolar wall. To limit the measurements to alveolar parenchyma, areas containing airways or blood vessels 25 *μ*m in diameter was excluded from the analysis. Average thickness of the Sirius Red-positive connective tissue of the alveolar wall was computed from two times the ratio of volume density of point to the surface density of the alveolar wall.

#### 2.7.2. Si NW Distribution and Clearance

Tissue sections from left lung of control rats and rats treated with 100 *μ*g Si NW were stained with Sirius red and hematoxylin as described above. The same point intercept counting method described above was used for volume of Si NW in the alveolar region to determine total lung burden of Si NW as a percentage of the total burden on day 1 after-instillation as a measure of lung clearance over time. In addition, point counting categories for Si NW in airways and in the alveolar region were evaluated. Airway regions were defined as those containing airway tissue (airway epithelial cells-basement membrane and tissues of the bronchovascular cuff), airway lumen, and associated blood vessels greater than 25 microns. Alveolar regions were those containing alveolar tissue, alveolar macrophages, and alveolar air space, for which the distribution in each alveolar region was also measured.

### 2.8. Statistical Analysis

Results for toxicity studies and morphometric studies were expressed as means ***±*** standard errors, and an analysis of variance (ANOVA) was performed to determine significant difference among treatment groups. For toxicity studies, significant differences among groups were assessed by the Student-Newman-Keuls method. For morphometric studies, Bartlett’s test was used to test for homogeneity of variances between groups. When significant *F* values were obtained, individual means were compared to control using Duncan’s multiple comparison procedure. Because data from histopathology studies were inherently categorical, a nonparametric analysis of variance was performed using SAS, Inc. statistical programs using the Wilcoxon rank sum test. For all analyses, significance was set at *P <* 0.05.

## 3. Results

### 3.1. Si NW Physical Characterization

**Figure 1:**
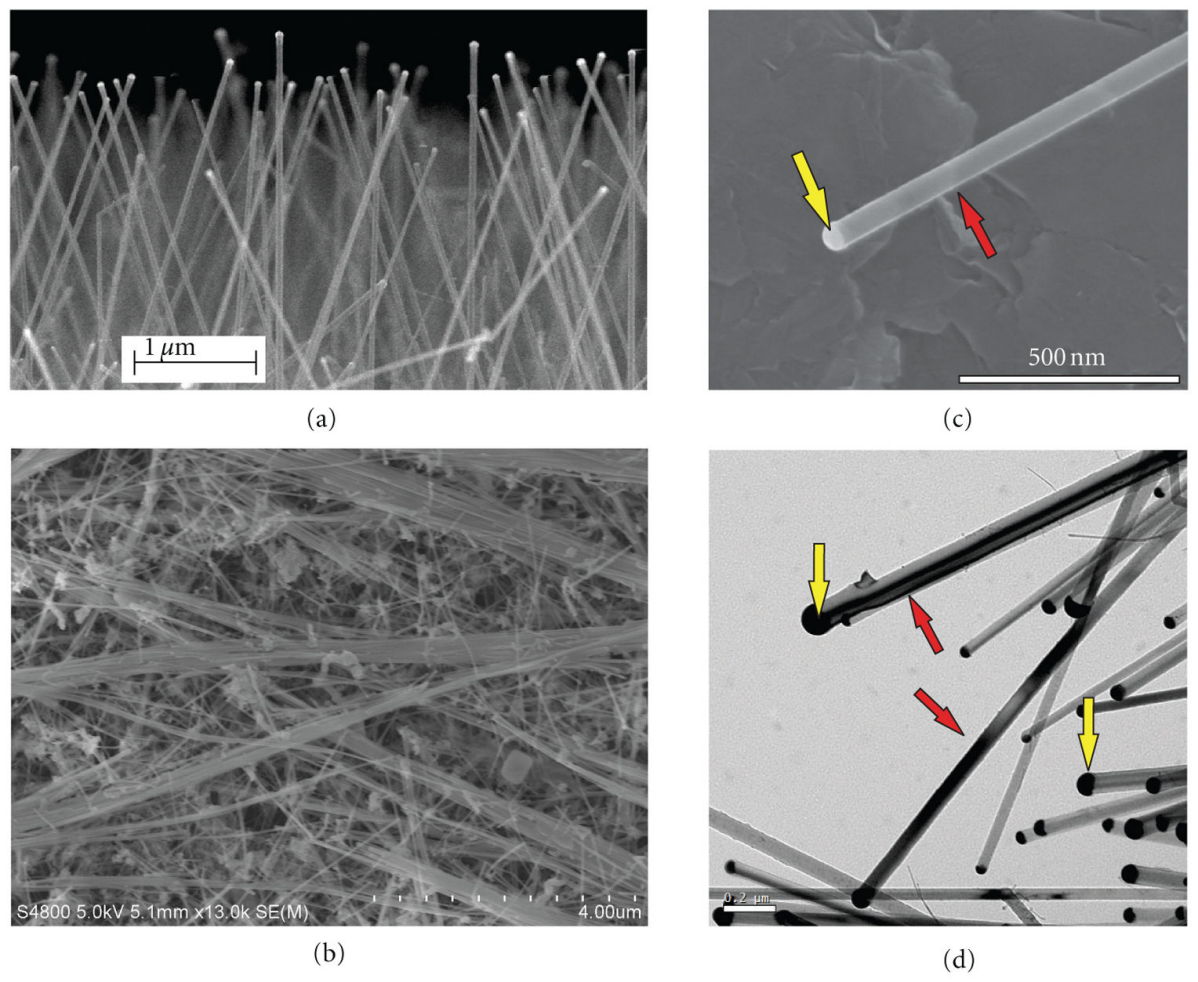
Field emission scanning electron microscopy (FESEM) image of silicon nanowires (Si NW) grown on a wafer using a vapor-liquid-solid (VLS) method with silane as the silicon precursor and 25 nm gold nanoparticles as the catalyst (a). FESEM image of concentrated Si NW that had been removed from the wafer and dried onto a planchet (b). A FESEM micrograph (c) and a transmission electron micrograph (d) showing individual Si NW (red arrows) with gold catalyst nanoparticles (yellow arrows) at one end of the wire after the Si NW had been isolated from the wafer.

FESEM was used to provide images of Si NW used in the current toxicology study (Figure 1). Si NWs were grown on Si(111) wafers using agglomerated gold nanoparticles as catalysts (Figure 1(a)), removed from the Si wafers before physical characterization and cellular/animal treatment, and dried onto planchets (Figure 1(b)). A FESEM micrograph (Figure 1(c)) and a transmission electron micrograph (Figure 1(d)) showed individual Si NW (red arrows) with gold catalyst nanoparticles (yellow arrows) at one end of the wire after the Si NW had been isolated from the wafer. The gold content in the Si NW was determined by neutron activation to account for ~10% of the sample by weight. Si NWs also have native SiO_2_ along their surface as a product of contact with the environment. ESR showed that surface reactivity was slightly higher in the samples that contained the gold catalyst versus the Si NW samples that had the catalyst removed; however, there was very little surface reactivity in either of the Si NW samples relative to the positive control particle that had a high surface reactivity (data not shown). Why little reactivity was measured is likely due to the SiO_2_ on the surface of the Si NW rather than the wire itself.

**Figure 2:**
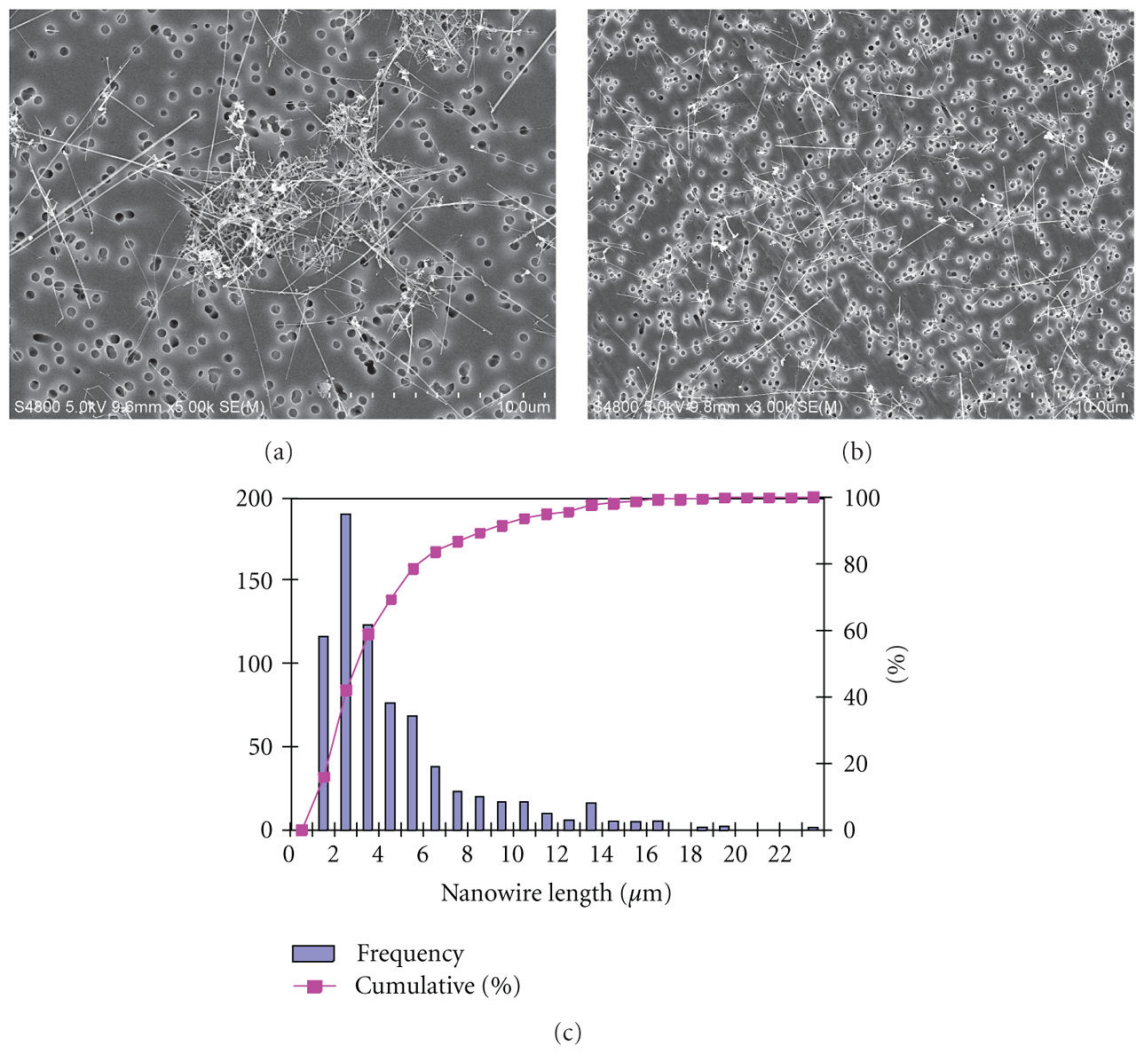
Field emission scanning electron micrographs of Si NW suspended and sonicated in phosphate-buffered saline (a) or dispersion medium (b) showing that dispersion medium effectively diminished agglomeration of wires. Electron micrographs of Si NW in dispersion medium were used to establish the length distribution of the NW. A histogram was generated from 730 length measurements of Si NW (c) (frequency, left axis; cumulative percent, right axis). The majority of Si NW were found to fall in the range of ≤5 *μ*m in length while 30% was *>*5 *μ*m.

Si NWs were found to form rope-like bundles when suspended in aqueous PBS medium (Figure 2(a)). This formation of agglomerates was effectively diminished when suspending the Si NW in DM then sonicating (Figure 2(b)). A histogram of the length distribution of Si NW was determined from measures of 730 nanowire lengths. After removal from the wafer, 70% of the Si NW was found to fall in the range of *<*5 *μ*m in length and 30% was *>*5 *μ*m. Breakage of the NW was found to occur due to removal from the wafer and from further sonication in DM.

### 3.2. In Vitro Uptake by Primary Alveolar Macrophages (AMs)

**Figure 3:**
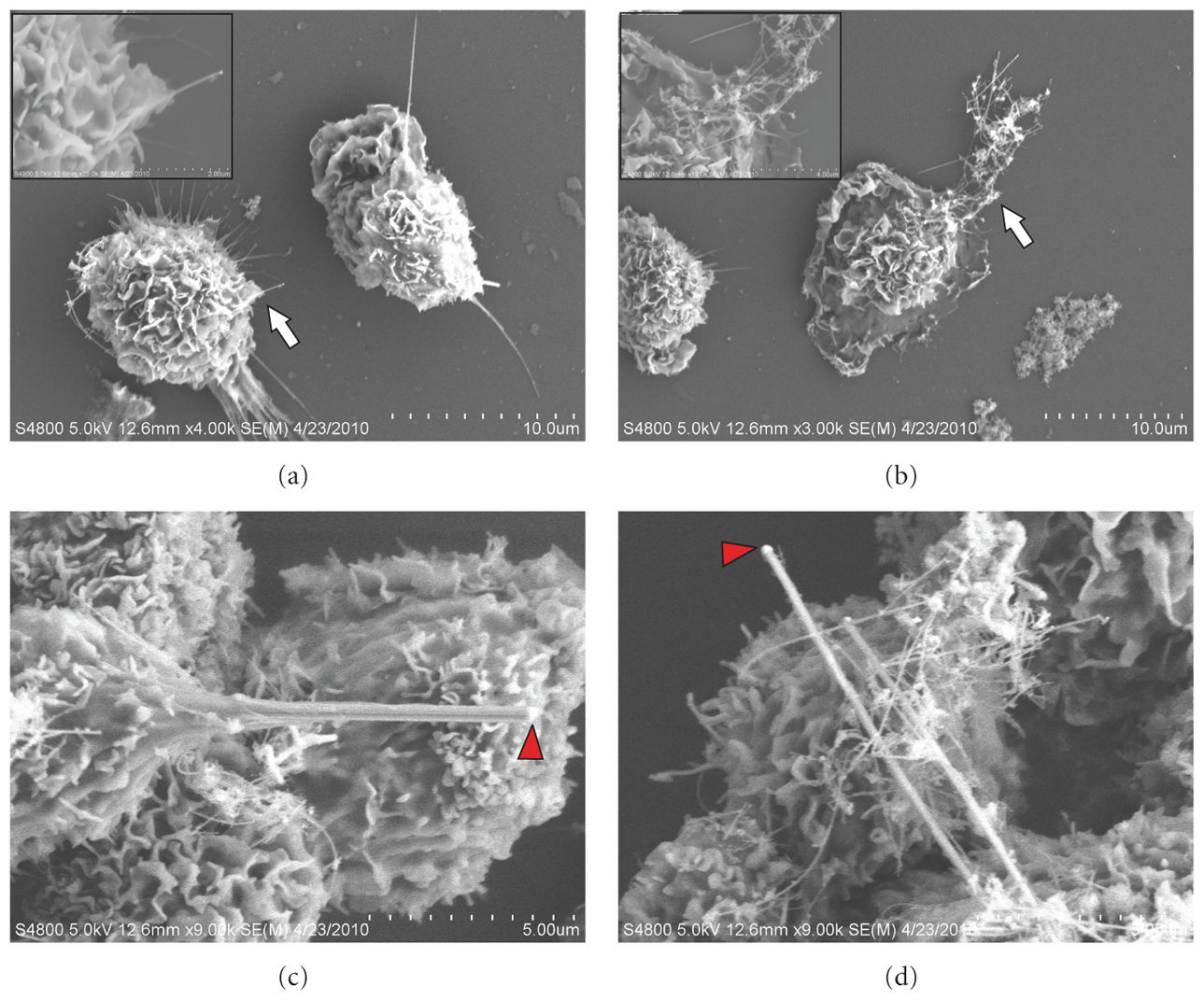
Low magnification (a) and (b) and high magnification (c) and (d) field emission scanning electron micrographs of Si NW uptake by primary rat alveolar macrophages (AMs) *in vitro* after a 4-hour incubation with 25 *μ*g of Si NW. AMs readily scavenged and engulfed both individual Si NW (a) as well as agglomerates of Si NW (b) (area of inset indicated by white arrows). The gold catalyst particle can also be seen at high magnification (red arrow head, (c) and (d)).

Naïve AMs recovered from untreated rats readily scavenged and engulfed both individual NW (Figure 3(a), white arrow) as well as agglomerates of NW (Figure 3(b), white arrow). The gold catalyst nanoparticle can be observed at the tip of the wire after a 4 hr incubation with the AMs (Figures 3(c) and 3(d), red arrowhead).

### 3.3. In Vivo Pulmonary Injury and Inflammation

**Figure 4:**
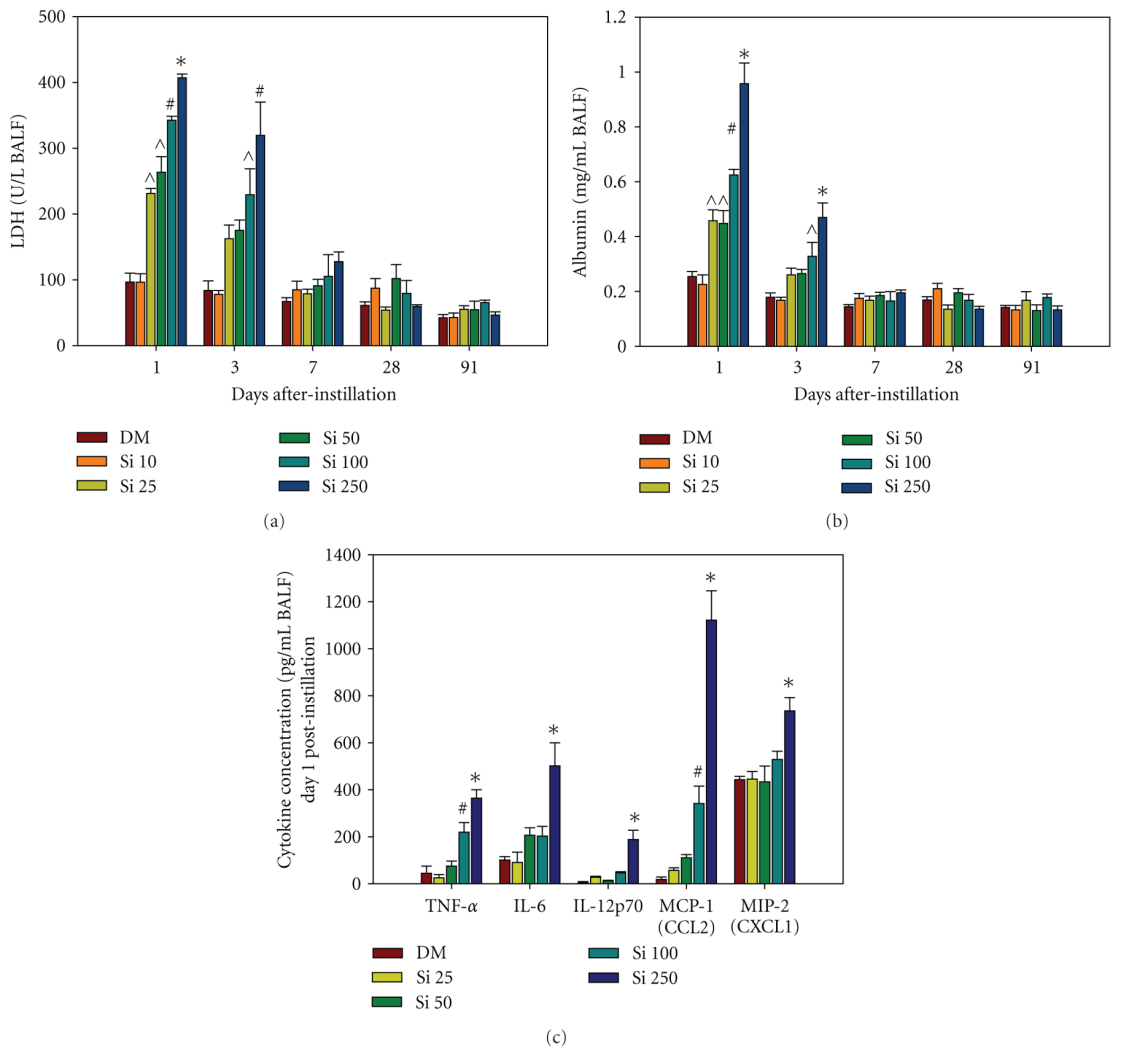
Indices of lung injury, lactate dehydrogenase (LDH) (a) and albumin (b), and inflammation, specific cytokines (c), were measured in the first fraction of BAL fluid at 1, 3, 7, 28, and 91 days after-intratracheal instillation with 10 (Si 10), 25 (Si 25), 50 (Si 50), 100 (Si 100), or 500 (Si 500) *μ*g Si NW, or dispersion medium control (DM). *significantly greater than all groups within a time point; ^#^significantly greater than DM, Si 10, Si 25, and Si 50; ^^^significantly greater than DM and Si 10; values are means ***±*** standard error (*n* ***=*** 8 DM, *n* ***=*** 4 Si NW; *P <* 0.05).

For the measurement of lung injury in BALF, there was a significant dose-dependent increase in LDH (Figure 4(a)) and albumin (Figure 4(b)) at 1 and 3 days after Si NW treatment compared to DM control. There were no significant increases in indices of lung injury at 7 days after treatment with any of the doses of Si NW. Proinflammatory cytokines (TNF-*α*, IL-6, and IL-12p70) and chemokines (MCP-1 and MIP-2) were significantly elevated only at 1 day after the highest dose of Si NW treatment compared to DM control (Figure 4(c)). No differences were observed between the Si NW and DM groups when measuring TGF-*β*, IL-2, IL-4, IL-10, and IFN-*γ* in the BALF (data not shown).

**Figure 5:**
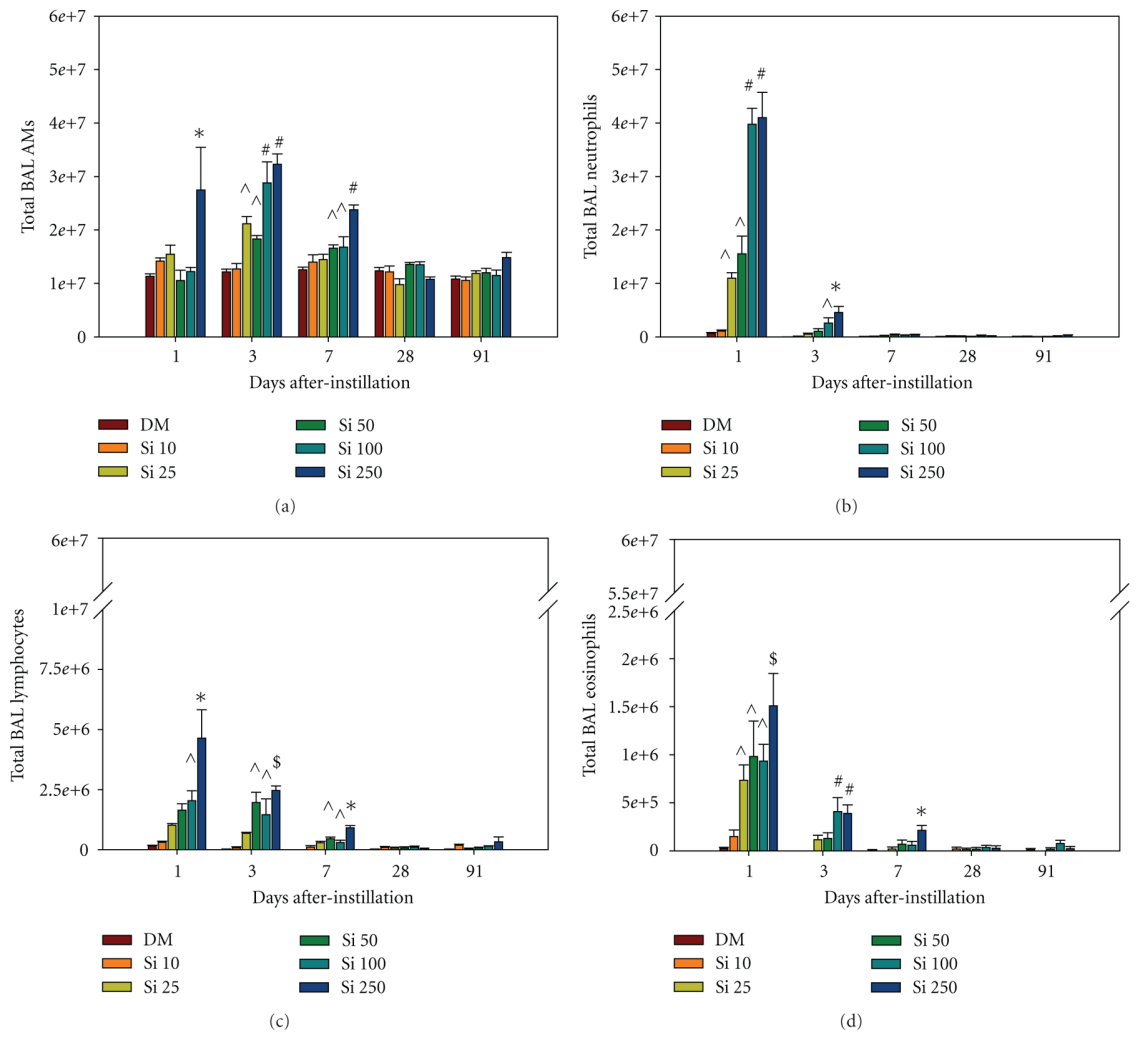
Total number of (a) alveolar macrophages (AMs), (b) neutrophils, (c) lymphocytes, and (d) eosinophils recovered by BAL from rats 1, 3, 7, 28, and 91 days after intratracheal instillation with 10 (Si 10), 25 (Si 25), 50 (Si 50), 100 (Si 100), or 500 (Si 500) *μ*g Si NW or dispersion medium control (DM). *significantly greater than all groups within a time point; ^#^significantly greater than DM, Si 10, Si 25, and Si 50; ^$^significantly greater than DM, Si 10, and Si 25; ^^^significantly greater than DM and Si 10; values are means ***±*** standard error (*n* ***=*** 8 DM, *n* ***=*** 4 Si NW; *P <* 0.05).

**Figure 6:**
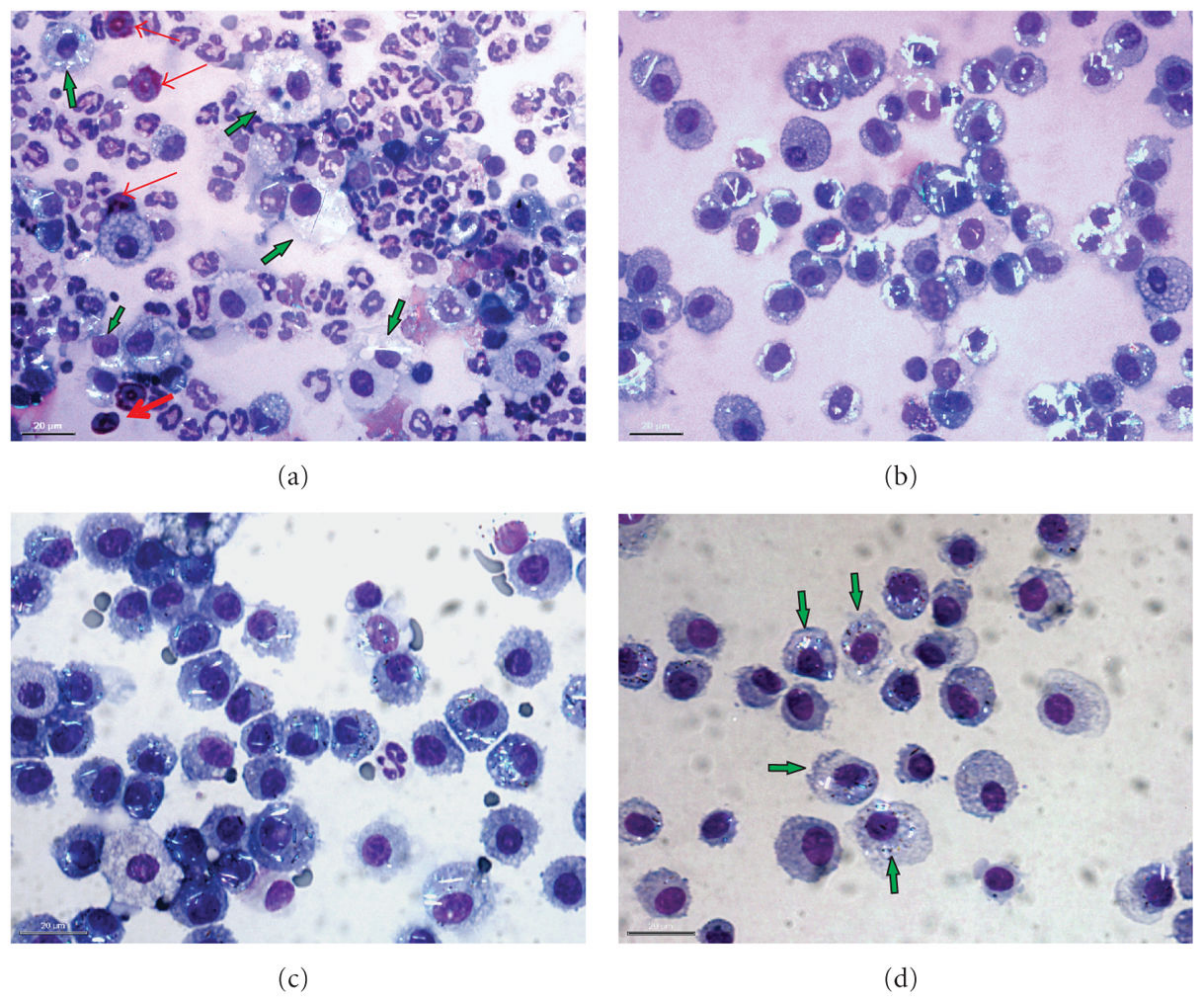
Cytospins of BAL cells recovered from rats on days 1 (a), 7 (b), 28 (c), and 91 days (d) after intratracheal instillation with 250 *μ*g of Si NW, demonstrating resolution of inflammatory response over time. 1 day after instillation of Si NW, there is a strong inflammatory response with significant neutrophil influx, as well as eosinophil influx (red arrows). AMs containing the Si NW (shiny birefringent material) indicated by the green arrows decreased over time. Additionally, reduction of Si NW burden in AMs over time was observed. See Table 3 and Figure 10 regarding Si NW clearance from the lung.

**Figure 7:**
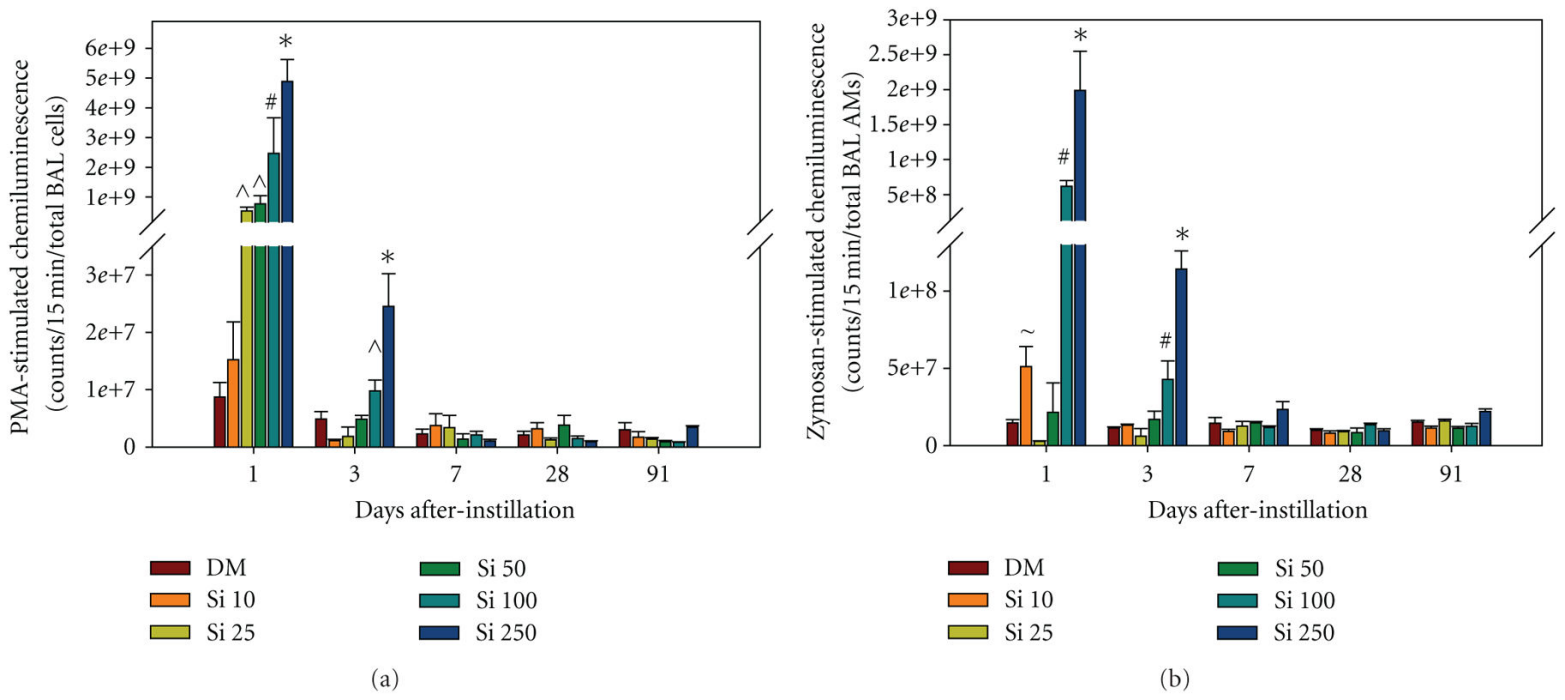
Chemiluminescence, an index of oxidant production, was measured in cells recovered by BAL from rats 1, 3, 7, 28, and 91 days after intratracheal instillation with 10 (Si 10), 25 (Si 25), 50 (Si 50), 100 (Si 100), or 500 (Si 500) *μ*g Si NW or dispersion medium control (DM): (a) total phagocyte (neutrophils and AMs) chemiluminescence depicted as total counts per 15 min for total BAL cells after stimulation with phorbol 12-myristate 13-acetate (PMA), (b) AM chemiluminescence depicted as total counts per 15 min for total BAL AMs after stimulation with nonopsonized zymosan. *significantly greater than all groups within a time point; ^#^significantly greater than DM, Si 10, Si 25, and Si 50; ^~^significantly greater than DM and Si 25; ^^^significantly greater than DM and Si 10; values are means ***±*** standard error (*n* ***=*** 8 DM, *n* ***=*** 4 Si NW; *P <* 0.05).

For the measurement of cellular inflammation, lung cells recovered by BAL were counted and differentiated (Figure 5). AMs were significantly increased at 1 day after treatment with the highest Si NW dose and at 3 and 7 days after treatment with the higher Si NW doses compared to DM control (Figure 5(a)). There was a significant dose-dependent increase in neutrophils on days 1 and 3 after treatment with the Si NW compared to DM control (Figure 5(b)). In addition, there was a dose-dependent increase in both lymphocytes (Figure 5(c)) and eosinophils (Figure 5(d)) that last persisted for 7 days after treatment with Si NW. There were no significant differences in any of the types of recovered BAL cells at days 28 and 91 when comparing the Si NW and DM groups. Figure 6 illustrates representative cytospins of BAL cells recovered from rats on days 1 (Figure 6(a)), 7 (Figure 6(b)), 28 (Figure 6(c)), and 91 (Figure 6(d)) exposed to 250 *μ*g of Si NW. At 1 day after instillation of Si NW, the strong inflammatory response with significant neutrophil influx, as well as eosinophil influx (red arrows), can be seen. AMs that contained Si NW (shiny birefringent material) indicated by the green arrows, and the amount of Si NW in AMs, decreased over time. The phagocytes that infiltrate the lungs early after Si NW exposure were primed to produce increased oxidants as measured by CL *ex vivo*, an index of reactive oxidant species generation in AMs and neutrophils recovered by BAL (Figure 7). Dose-dependent increases in PMA-stimulated CL (Figure 7(a)) and zymosan-stimulated CL (Figure 7(b)) were observed at 1 and 3 days after treatment with Si NW. No significant changes in CL were seen at 7, 28, and 91 days after treatment when comparing the Si NW and DM groups. The CL response closely resembled the cellular inflammatory response that was observed with the cell differentiation analyses.

Histological evaluation of lung tissue was also performed to evaluate lung injury and inflammation. There was a dose-dependent increase in the parameters indicative of inflammation (perivascular monocyte infiltrates, alveolar macrophage and neutrophil aggregates, and pneumonia) and irritation (perivascular and peribronchiolar eosinophil infiltrates) at the early time points after-exposure, with the highest average score on the severity scale of 2 considered to be mild (Table 1). Inflammation was primarily located in the parenchyma surrounding terminal bronchioles. Bronchiolar degeneration/regeneration, an additional measure of injury and inflammation, was also increased at the highest doses early after exposure. Degeneration was characterized by occasional karyorrhexis, as well as infiltration by granulocytes. Regenerative changes were characterized by focal hyperplasia and rare mitoses. These changes occurred primarily at the junction between the terminal bronchiole and the alveolar duct. No significant changes in the histopathological evaluation of inflammation and injury were found on day 28 and 91 after-exposure.

**Figure 8:**
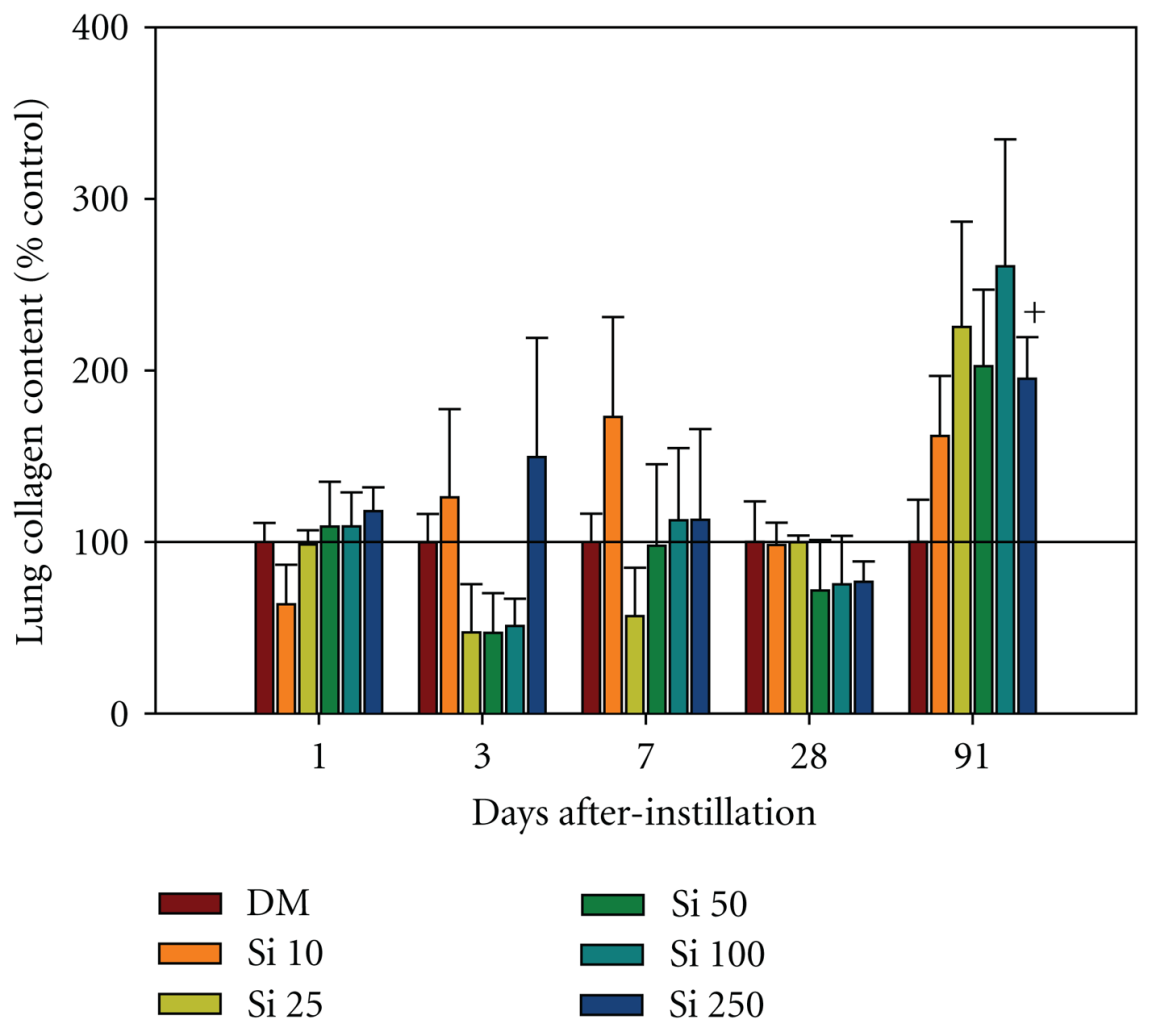
Collagen content measured by the sircol assay in the tissue of the right lungs after lavage was performed in rats 1, 3, 7, 28, and 91 days after intratracheal instillation with 10 (Si 10), 25 (Si 25), 50 (Si 50), 100 (Si 100), or 500 (Si 500) *μ*g Si NW or dispersion medium control (DM). ^+^significantly greater than DM; (*n* ***=*** 8 DM, *n* ***=*** 4 Si NW; *P <* 0.05).

In addition to the histopathological analysis, morphometric and biochemical analysis of lung collagen was performed to evaluate the potential of Si NW to induce fibrosis. In the morphometric analysis of alveolar wall thickness as a result of treatment with 100 *μ*g of Si NW, the percentage of alveolar wall that was connective tissue and the average thickness of alveolar wall connective tissue were significantly increased at days 28 and 91 after-instillation compared to DM control (Table 2). Biochemical analysis of collagen content by the sircol assay showed a trend for increased collagen content in the lungs of rats treated with Si NW at 91 days post-exposure with a significant increase of collagen in the 250 *μ*g dose of Si NW when compared to control rats (Figure 8). Although there is evidence of increased collagen and alveolar wall thickness at the later times after-exposure, histopathological evaluation of lung tissue resulted in no observations of fibrosis (Table 1).

### 3.4. Lung Tissue Distribution and Clearance

**Figure 9:**
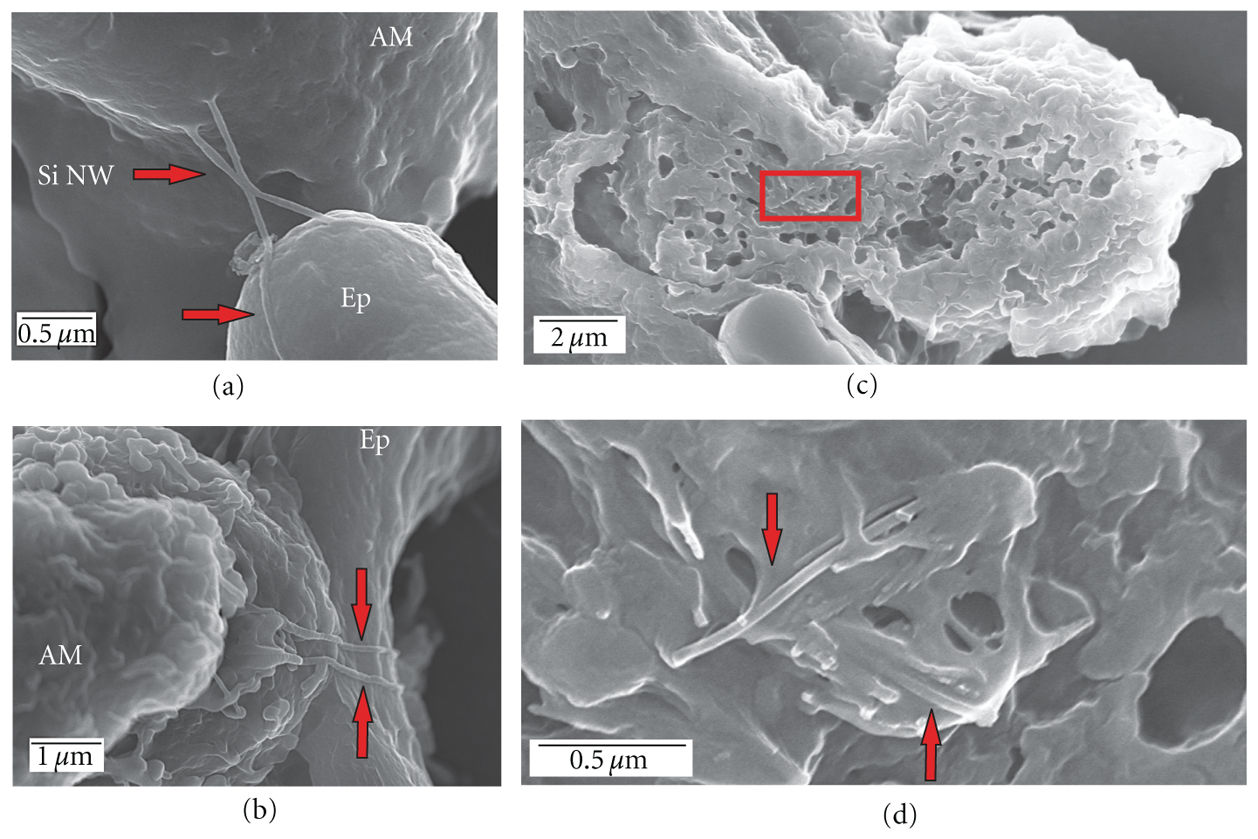
Field emission scanning electron micrographs of lung tissue from rats intratracheally instilled with 100 *μ*g Si NW 1 (a), 7 (b), 28 (c) and (d) days after-exposure. At 1 and 7 days, AMs can be seen engulfing Si NW (red arrows) from the epithelial lining (Ep) of the alveoli (d) is a high magnification of the area in the red box in (c), illustrating an interaction between an AM and multiple Si NW 28 days after-exposure.

FESEM was used to examine Si NW *in situ* at different time points after pulmonary treatment (Figure 9). At days 1 (Figure 9(a)) and 7 (Figure 9(b)) after treatment, AMs can be seen engulfing Si NW (red arrows) from the epithelial lining (Ep) of the alveoli. Also at 28 days after treatment, interactions between AMs and multiple Si NW were observed (Figures 9(c) and 9(d)).

**Figure 10:**
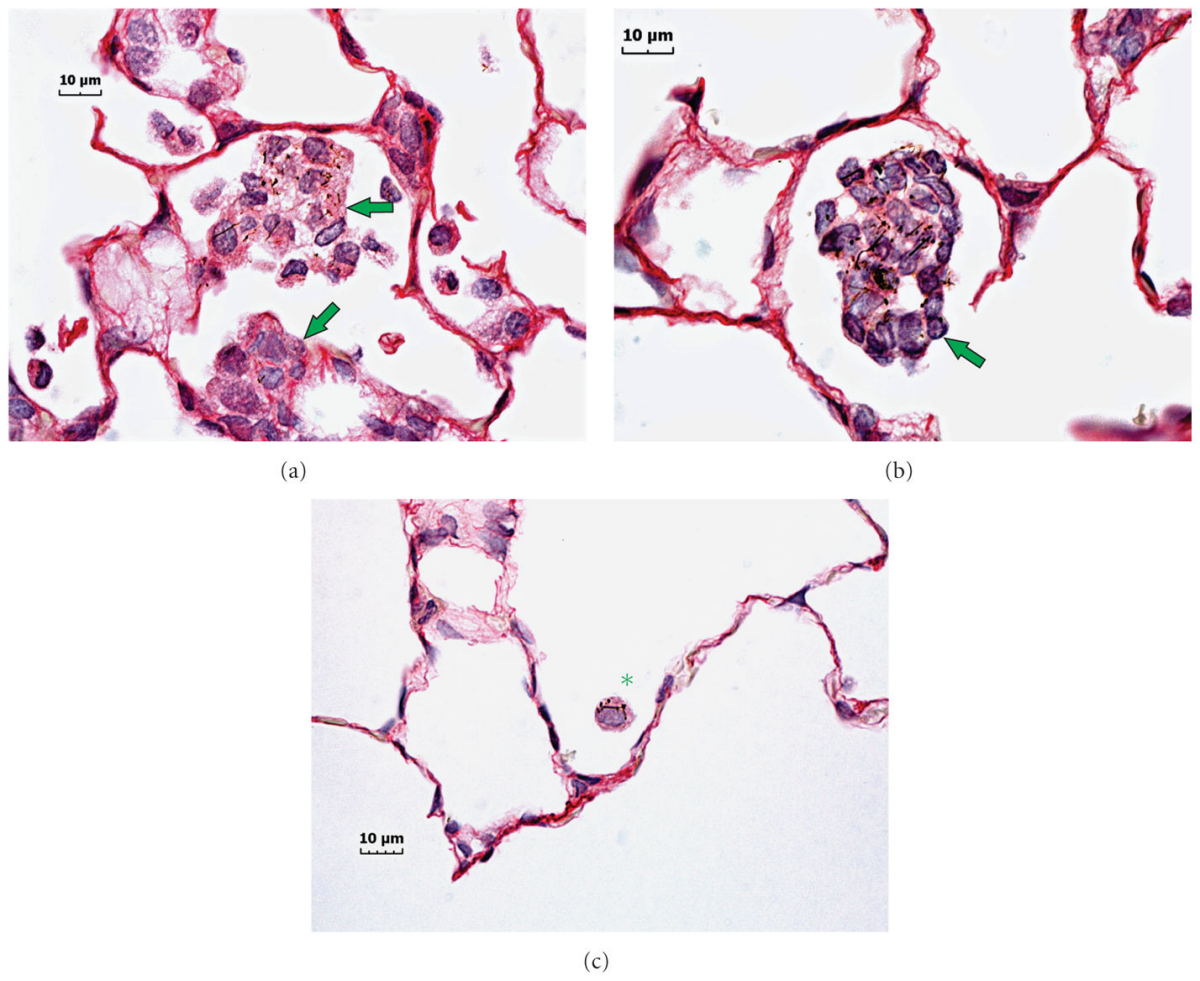
Sirius red-stained tissue sections from the left lungs of a rat intratracheally instilled with 100 *μ*g of Si NW 3 (a) and (b) and 91 (c) days after-exposure. 3 days after-exposure granulomatous-type lesions consisting of macrophages containing Si NW are readily observed (green arrows). By day 91 the alveoli are relatively clear, and the Si NW present are located in AMs (green asterisk).

**Figure 11:**
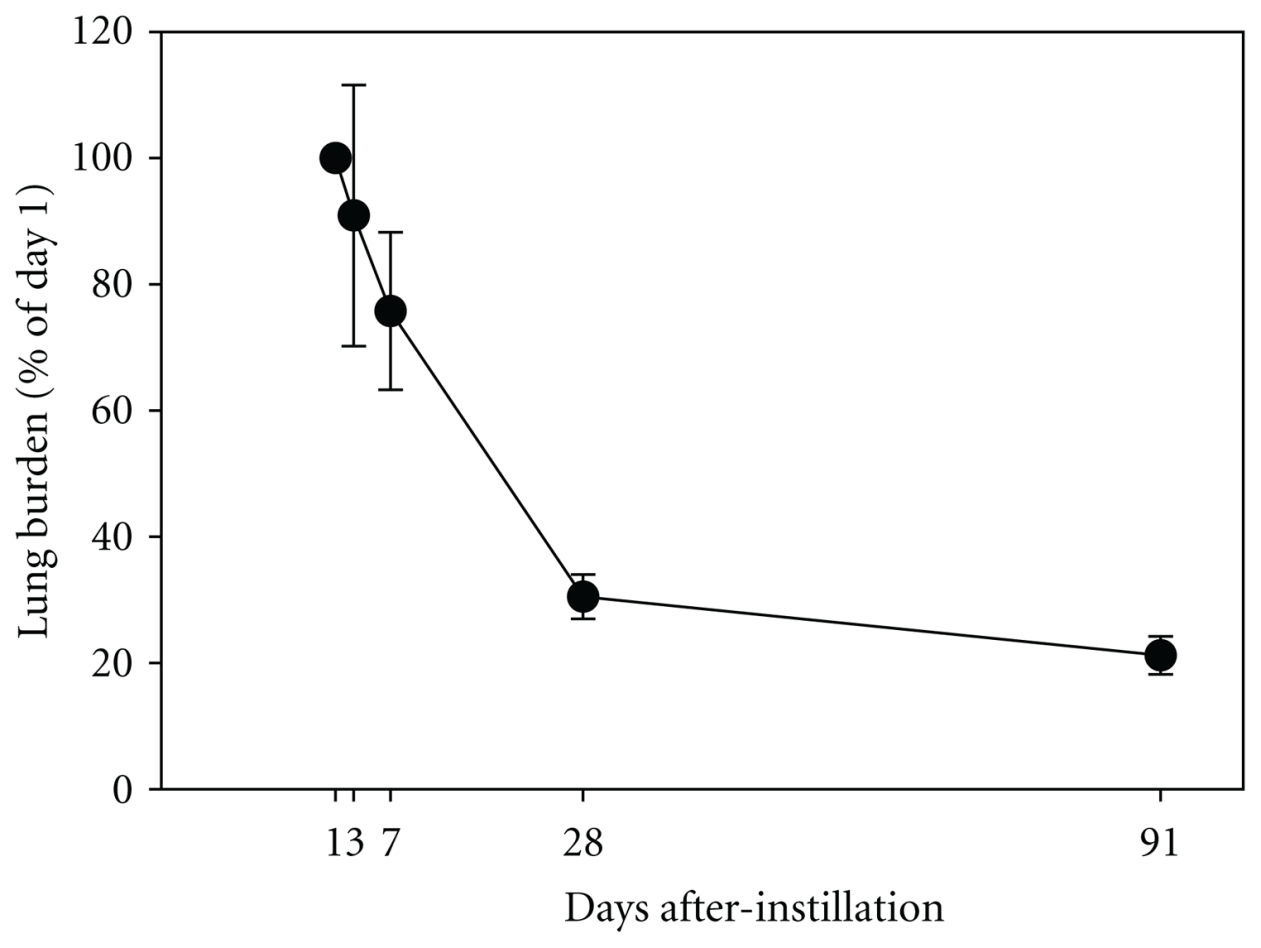
Morphometric measurement of Si NW lung burden in rats treated with 100 *μ*g Si NW. Pulmonary clearance of Si NW is presented as the percent of the burden on day 1 after-instillation.

Sirius red-stained tissue sections from the left lungs were examined for lung histopathology and the deposition of Si NW (Figure 10). At 3 days after Si NW treatment with 100 *μ*g dose, granulomatous-type lesions were observed throughout the alveoli. The lesions consisted of AMs that contained Si NW (Figures 10(a) and 10(b), green arrows). By day 91, the alveoli were relatively clear of lesions and any Si NW that remained in the lungs were observed to reside exclusively in AMs (Figure 10(c), asterisk). Morphometric analysis indicated that approximately 70% of the deposited Si NW was cleared from the lungs by day 28 after treatment (Figure 11). The clearance rate slowed after day 28, and ***~***20% of the burden remained at day 91. No Si NWs were observed in the airways at 1 day after treatment with 100% of the burden located in the alveolar region (Table 3). At 28 and 91 days after treatment, the Si NWs were localized exclusively in the AMs.

## 4. Discussion

Pulmonary toxicity associated with high aspect ratio fibers and nanomaterials is likely caused by a number of different factors that can induce lung inflammation and disease including chemical composition, surface reactivity of the material, solubility, physical dimensions, agglomeration state, and dose. The most common example of fiber-induced toxicity to which wire-shaped nanomaterials have been compared is that of amphibole forms of asbestos (e.g., crocidolite), where lung inflammation and fibrosis progress over time after exposure. In the case of asbestos and other manufactured fibers, lung fibrosis has been shown to be dependent on dose, dimension (length and width), and durability leading to biopersistence in the lung [29, 30]. These variables, commonly referred to as the fiber pathogenicity paradigm, lead to increased lung burden of fibers in the alveolar region of the lung and impede particle clearance there. Particles less than 3 *μ*m in width deposit more readily in the respirable region of the lung; long fibers with lengths greater than 15 *μ*m frustrate phagocytosis and clearance by AMs; particles that do not dissolve or break are not readily cleared from the lungs, and overload doses affect AM mobility and slow lung clearance [2]. The biopersistence of the fibers in the alveolar region then leads to progression of inflammation and oxidative stress and the eventual development of fibrosis in both the lung and pleura.

In regards to nanomaterials following this paradigm, the pathogenicity pattern observed with one of the better studied high aspect ratio nanomaterials, CNT, has been compared to that of asbestos [31]. *In vivo* studies of pulmonary toxicity associated with SWCNT and MWCNT have varied in relationship to rodent species and strain used, dose, particle purity, surface chemistry, composition, particle size and dimension, and agglomeration state. With the exception of a minority of rodent studies, the pulmonary response after respiratory exposure to SWCNT [7, 11, 32] or MWCNT [6, 8, 9, 33] has been shown to be characterized by a dose-dependent increase in inflammation and oxidative stress leading to lung injury and fibrosis. Although inflammatory responses began to resolve at later time points after exposure, collagen deposition in the alveolar region and fibrosis were significant and did not resolve, beginning as early as 1 week after-exposure.

In the Si NW study presented here, inflammation resolved at earlier time points relative to that observed with CNT studies and increases in collagen content, measured as total collagen with the sircol assay, were not detected until 91 days after-exposure at the highest dose (250 *μ*g/rat), unlike the early-onset fibrosis observed after CNT exposures. Fibrosis was not found in histopathological analysis. With concern that the highest dose may be an overload burden, morphometric analyses of fibrillar collagen content and septal wall thickening were performed at the dose of 100 *μ*g/rat (equivalent to 0.4 mg/kg of body weight), and both were found to increase at days 28 and 91 after-exposure. Lung burden and clearance were also analyzed at this dose, and approximately 20% of the initial dose remained in the lungs at day 91, although the burden was contained entirely within AMs at this time. Granulomatous lesions were evident early but decreased over time. In contrast, CNT produced granulomatous lesions that persisted at least 56 days after-exposure. Comparing differences in Si NW and CNT responses, as a model high aspect ratio nanoparticle that has been associated with asbestos-like pathogenicity, and discerning whether these are due to differences in dose, length, durability, and/or composition, is complicated.

The doses used in the present study were relatively low compared to those in the literature that exist for intra-tracheal exposure to CNT in rats. The highest dose used in this study, 250 *μ*g per rat, equates to 1 mg/kg of body weight. In the study by Warheit et al. [32], SD rats were instilled with 1 or 5 mg/kg of body weight of SWCNT (primary size of 1.4 nm diameter and *>*1 *μ*m length before agglomeration). At the 1 mg/kg dose, there was little evidence of inflammation after 1 week, similar to the responses observed after treatment with the high dose of the Si NW study. However, granulomatous lesions did develop at 1 week after exposure to the 1 mg/kg SWCNT dose which either decreased or did not increase by 3 months after-exposure, unlike the 250 *μ*g Si NW dose in the present study where the only notable change was an increase in collagen without full development of fibrosis. Aiso et al. [33] examined MWCNT (80 nm in diameter ***×*** 5 *μ*m in length) after an intratracheally administered doses of 40 and 160 *μ*g per rat in F344 rats, doses that were comparable to those used in the current Si NW study. In addition, the length distribution in that study was similar to the size distribution of the current Si NW study where 70% of the Si NW was less than 5 *μ*m long. Pulmonary responses were followed up to 3 months after-exposure, and inflammation was found to be dose dependent and increased significantly in both doses at all time points after-exposure. In addition, fibrosis was found at the later time points beginning at day 28 in the high dose and at day 90 in the low dose. The pattern of pathogenesis differed greatly from the current study, leading to the assumption that differences may be due to composition and/or biopersistence of the CNT; however, the F344 strain of rat has proven to be more sensitive to inflammatory stimuli. In contrast, Kobayashi et al. [34] showed that intratracheal instillation of dispersed MWCNT (60 nm ***×*** 1.5 *μ*m in size) in SD rats, at doses ranging from 0.04 to 1 mg/kg body weight (dispersed in Tween 80), showed only a transient dose-dependent increase in pulmonary inflammation with no chronic pathogenesis up to 6 months post-exposure. In this particular study, the rat strain, dose, time points, and particle dimensions were similar to the Si NW study, with more comparable findings; however, the investigators did not observe increased collagen deposition as was seen in the Si NW study. Possible reasons for this difference include different clearance patterns of the CNT versus Si NW over time or differences in biopersistence at later time points.

As mentioned earlier, the majority of findings regarding CNT are not in agreement with those of Kobayashi et al. [34] and the general consensus is that exposure induces inflammation and fibrosis. In mouse aspiration studies, SWCNT [11] and MWCNT [8], which contained equivalent doses of CNT by weight to the Si NW rat study reported here, induced inflammation that persisted up to 1 month after-exposure with the development of persistent fibrosis that began at 1 week after-exposure. In the study by Shvedova et al. [11], lower aspiration doses of 10 and 20 *μ*g per mouse were fairly comparable upon normalizing to body weight to the 100 and 250 *μ*g doses in the rats treated with Si NW. Inflammation was observed at these lower doses of SWCNT, decreasing over time but persisting for up to 2 months after-exposure. Fibrosis developed early with a dose as low as 20 *μ*g and continued to progress over time. Unfortunately, average agglomerate size in the aspirate was not available for length comparison. Interestingly, in the study by Porter et al. [8], the length distribution and relative width of the dispersed MWCNT samples, with median length of 3.86 *μ*m and width of 49 nm, were very similar to the bulk of the dispersed Si NW sample. In that study, mouse aspiration doses as low as 10 *μ*g per mouse (comparable to the 100 *μ*g dose of Si NW in the rat) produced an inflammatory response that persisted up to 1 month after-exposure with on onset of fibrosis at 1 week after-exposure, which persisted through the 56 day time course, a pattern very different from that observed in the Si NW study presented here.

It is important to note that in discussing dose comparison in terms of weight, it needs to be considered that the number of particles, as either individual particles or agglomerates, will vary depending on the composition of the nanomaterial. CNTs are lighter in weight than Si NW. For the studies discussed above that included equivalent doses by weight, a far greater number of CNT rope-like agglomerates would have been present for a given dose, which may account for differences observed in the pathogenicity profile. Comparisons based on particle number are difficult to assess. In the literature, the majority of studies on the effects of nanomaterials in the lung using instillation or aspiration as a route of administration have been presented in terms of material weight. Although surface area of dry material and primary particle dimension is usually included in material characterization, which would allow for an estimate of particle number, effective particle number in solution after agglomeration or aggregation differs greatly from study to study depending on solution preparation, whether the materials have been dispersed, and type of dispersant used [35]. Agglomeration variables in turn also affect the size distribution and effective length and width of the agglomerates. As noted in the studies discussed above, where primary sizes of CNT are generally smaller in both dimensions relative to Si NW, the agglomerates were more comparable to the size distribution of the Si NW sample used in the current study.

Although length of CNT has been shown to play a definitive role in toxicity [36, 37], it is interesting to note that in terms of the fiber pathogenicity paradigm, the CNT samples in the studies discussed above are shorter than the 15 *μ*m length dimension associated with frustrated phagocytosis. Relative to dose and length, at least with regard to the shorter fraction of the Si NW sample accounting for the majority of the sample, the studies discussed above suggested a greater potency of CNT, which may depended on variables other than dose and length, such as factors related to composition, including surface chemistry and reactivity. Multiple studies have indicated an association that relates compositional differences and differences in surface chemistry of various SWCNT and MWCNT samples, such as the presence of metals and free radical production, with enhanced potential for inducing oxidative stress, as reviewed by Johnston et al. [35]. The surface reactivity of Si NW samples (with and without the gold catalyst) used in the current study was evaluated in an acellular system using ESR. Free radical signals associated with the surface of the Si NW in absence of gold were not significantly elevated and the sample used in this study which contained gold had only a slight increase in reactivity, as gold is among the more stable of the transition metals. Relative to *α*-quartz, a highly reactive positive control sample, and despite the associated SiO_2_ that forms on the surface of the nanowires, there was no significant generation of free radicals and hence little surface reactivity. *In vivo*, oxidant production by AMs also returned to control levels within 1 week of exposure to Si NW, unlike what was observed with particles that have high surface reactivity, such as *α*-quartz or certain forms of CNT.

Whether increases in inflammation and oxidant production are attributable to the small fraction of the sample that is gold (***~***25 *μ*g at the high dose) are debatable. There are presently very few studies that investigate the effects of nanogold on pulmonary toxicity. Of those that exist, a 90-day inhalation study at 20 *μ*g/m^3^ found a mild increase in pulmonary inflammation 1 day after exposure [38], and a study which examined toxicity after intratracheal instillation of 400 *μ*g of nanogold comparing two different sizes (50 and 250 nm in diameter) found only a slight but significant increase in inflammation with the larger of the two 1 day after exposure [39]. In the current study, it is possible that gold is contributing to the inflammatory response, but given the amount present, and incomparison to the two studies mentioned above, the Si portion or the wire shape are likely the greatest contributors.

Based on the particle distribution/clearance pattern and the resolution of inflammation and oxidant production early after exposure to Si NW, the potential causes of the increase in collagen observed biochemically and morphologically at 91 days after-exposure may be correlated to the dose in relationship to particle load in the lung and/or the potential persistence of the longer fraction of the instillate. The finding that the Si NW used in this study, which contained the gold eutectic at one end and SiO_2_ along the surface, was not very reactive, and that inflammation and oxidant production in the lung have subsided greatly by 1 week after-exposure when the lung burden was still around 70% indicate that composition may not be playing a critical role in the later response of increased collagen in the lung. In relationship to the fiber pathogenicity paradigm, several questions remain to be answered. The bulk of the sample was below the critical length for frustrated phagocytosis; however, a portion of the sample did fall into that length range where clearance can become hindered. Whether the longer fraction is the portion remaining at day 91 contributing to increased collagen in response to biopersistence, or whether this is related to persistence as a function of load remains to be elucidated. In addition, the durability of the Si NW in the lung or whether longer Si NW can be broken down and cleared remains to be assessed. A side-by-side comparison of samples that are uniformly different in length is necessary to determine whether longer Si NWs pose a potential health hazard.

## Figures and Tables

**Table 1:** Histopathological evaluation of lung tissue.

Histopathology parameter	Time after-instillation				
	Day 1	Day 3	Day 7	Day 28	Day 91
Perivascular monocyte infiltrates	Si 10 (0.50) Si 25 (0.75) Si 50 (1.00)+ Si 100 (1.00)+ Si 250 (1.25)+	Si 50 (0.75) Si 100 (1.00)^ Si 250 (2.00)*	Si 250 (1.00)*	—	—
Perivascular & peribronchiolar eosinophil infiltrates	Si 10 (0.50) Si 25 (1.25)^ Si 50 (1.50)^ Si 100 (1.25)^ Si 250 (1.75)^	Si 25 (0.50) Si 50 (0.75) Si 100 (1.00)^ Si 250 (1.25)^	Si 50 (0.50) Si 100 (1.00)~ Si 250 (1.00)~	Si 100 (0.50)	—
Interstitial pneumonia	Si 10 (0.50) Si 25 (1.25)+ Si 50 (1.50)+ Si 100 (1.50)+ Si 250 (2.75)*	Si 50 (0.75)+ Si 100 (0.75)+ Si 250 (2.25)*	—	—	—
Alveolar macrophage aggregates	Si 25 (1.00)^ Si 50 (1.50)^ Si 100 (1.25)^ Si 250 (2.00)$	Si 50 (1.00)+ Si 100 (1.00)+ Si 250 (2.00)*	Si 100 (0.50) Si 250 (1.00)$	Si 100 (0.50)	—
Alveolar neutrophil aggregates	Si 50 (0.75) Si 250 (0.50)	Si 100 (0.50) Si 250 (1.00)	—	—	—
Bronchiolar degeneration/regeneration	Si 25 (0.75)+ Si 50 (1.25)^ Si 100 (1.00)^ Si 250 (1.00)^	Si 50 (1.00)$ Si 250 (1.25)$	—	—	—
Fibrosis	—	—	—	—	—

Histopathology parameters were scored for severity on a scale of 0–5 where indices of inflammation and injury were graded as 0 (not present), 1 (minimal), 2 (mild), 3 (moderate), 4 (marked), or 5 (severe). Groups were included in a category when the parameter was observed in at least half of the animals in that group. The mean severity score is presented in parenthesis after the group (*n* = 4).

* significantly greater than all groups;

$ significantly greater than DM, Si 10 and Si 25;

~ significantly greater than DM and Si 25;

^ significantly greater than DM and Si 10;

+ significantly greater than DM; (*P <* 0.05).

**Table 2:** Morphometric analysis of connective tissue in rats treated with 100 *μ*g of Si NW.

Treatment group	% of Alveolar wall	Thickness (*μ*m)
DM Control	3.0 ***±*** 0.3	0.075 ***±*** 0.089
Si 100 Day 1	3.4 ***±*** 0.3	0.084 ***±*** 0.064
Si 100 Day 3	3.5 ***±*** 0.4	0.083 ***±*** 0.009
Si 100 Day 7	3.9 ***±*** 0.3	0.095 ***±*** 0.009
Si 100 Day 28	4.4 ***±*** 0.4*	0.110 ***±*** 0.012*
Si 100 Day 91	5.4 ***±*** 0.4*	0.135 ***±*** 0.010*

Note:

* significantly elevated when compared to DM control group, *P <* 0.05.

**Table 3:** Morphometric analysis of Si NW tissue distribution in rats treated with 100 *μ*g of Si NW.

Lung region	Time after-instillation				
	Day 1	Day 3	Day 7	Day 28	Day 91
Alveolar macrophage (AM)	72.37 ***±*** 3.61	86.46 ***±*** 5.21	90.63 ***±*** 5.98	100 ***±*** 0.00	100 ***±*** 0.00
Alveolar tissue (AT)	18.15 ***±*** 3.27	11.46 ***±*** 3.94	9.38 ***±*** 5.98	0.00 ***±*** 0.00	0.00 ***±*** 0.00
Alveolar airspace (AA)	9.47 ***±*** 3.22	2.08 ***±*** 2.08	0.00 ***±*** 0.00	0.00 ***±*** 0.00	0.00 ***±*** 0.00

Note: Values are percent of distribution of the remaining lung burden for a given time point. See Figure 10 for total lung burden and clearance rate.
